# Social stressors and air pollution across New York City communities: a spatial approach for assessing correlations among multiple exposures

**DOI:** 10.1186/1476-069X-13-91

**Published:** 2014-11-06

**Authors:** Jessie LC Shmool, Laura D Kubzansky, Ogonnaya Dotson Newman, John Spengler, Peggy Shepard, Jane E Clougherty

**Affiliations:** Department of Environmental & Occupational Health, University of Pittsburgh Graduate School of Public Health, 100 Technology Drive, Pittsburgh, PA 15219 USA; Department of Social and Behavioral Sciences, Harvard School of Public Health, 677 Huntington Avenue, Boston, MA 02115 USA; WE ACT for Environmental Justice (West Harlem Environmental Action), 1854 Amsterdam Avenue, New York, NY 10031 USA; Department of Environmental Health, Harvard School of Public Health, 401 Park Drive, Boston, MA 02215 USA

**Keywords:** Air pollution, GIS, Social stressors, Spatial confounding, Susceptibility

## Abstract

**Background:**

Recent toxicological and epidemiological evidence suggests that chronic psychosocial stress may modify pollution effects on health. Thus, there is increasing interest in refined methods for assessing and incorporating non-chemical exposures, including social stressors, into environmental health research, towards identifying whether and how psychosocial stress interacts with chemical exposures to influence health and health disparities. We present a flexible, GIS-based approach for examining spatial patterns within and among a range of social stressors, and their spatial relationships with air pollution, across New York City, towards understanding their combined effects on health.

**Methods:**

We identified a wide suite of administrative indicators of community-level social stressors (2008–2010), and applied simultaneous autoregressive models and factor analysis to characterize spatial correlations among social stressors, and between social stressors and air pollutants, using New York City Community Air Survey (NYCCAS) data (2008-2009). Finally, we provide an exploratory ecologic analysis evaluating possible modification of the relationship between nitrogen dioxide (NO_2_) and childhood asthma Emergency Department (ED) visit rates by social stressors, to demonstrate how the methods used to assess stressor exposure (and/or consequent psychosocial stress) may alter model results.

**Results:**

Administrative indicators of a range of social stressors (e.g., high crime rate, residential crowding rate) were not consistently correlated (rho = - 0.44 to 0.89), nor were they consistently correlated with indicators of socioeconomic position (rho = - 0.54 to 0.89). Factor analysis using 26 stressor indicators suggested geographically distinct patterns of social stressors, characterized by three factors: violent crime and physical disorder, crowding and poor access to resources, and noise disruption and property crimes. In an exploratory ecologic analysis, these factors were differentially associated with area-average NO_2_ and childhood asthma ED visits. For example, only the ‘violent crime and disorder’ factor was significantly associated with asthma ED visits, and only the ‘crowding and resource access’ factor modified the association between area-level NO_2_ and asthma ED visits.

**Conclusions:**

This spatial approach enabled quantification of complex spatial patterning and confounding between chemical and non-chemical exposures, and can inform study design for epidemiological studies of separate and combined effects of multiple urban exposures.

**Electronic supplementary material:**

The online version of this article (doi:10.1186/1476-069X-13-91) contains supplementary material, which is available to authorized users.

## Background

Within the field of environmental health, there is substantial interest in the combined effects of chemical and non-chemical exposures on human health [1–4]. Recent epidemiologic and toxicologic evidence indicates significant modification of pollution effects on health by chronic psychosocial stress [5–12]. For investigators interested in understanding the relationship between the social and physical environment, there is a growing need for refined, replicable methods for: a) measuring social stressor exposures across large cohorts, and b) reducing confounding between social and chemical exposures in environmental epidemiology [13].

Recent research on this topic has considered psychosocial stress as a possible key factor modifying the relationship between chemical exposures, including air pollution or lead, and adverse health outcomes [14]. Measurement of psychosocial stress differs from chemical pollution exposure assessment, because the physiologic impact of non-chemical stressors is mediated through individual perception [15]. Psychosocial stress is often a result of exposure to social stressors (i.e., an event, condition, or external stimuli posing a physical or psychological challenge). When individuals evaluate stressors as imposing demands that are beyond their ability to cope, a sense of distress results; with repeated exposure to such stressors this sense of distress can become chronic. Chronic psychosocial stress is associated with negative emotional states and maladaptive behaviors that influence immune, endocrine, and metabolic function to produce cumulative wear-and-tear – often referred to as *allostatic load* [16]. These physiologic changes may alter individuals’ reactivity to chemical exposures (e.g., pathogens, pollutants) and increase risk for multiple disease etiologies [17]. As such, individuals and communities who are chronically exposed to social stressors may be more susceptible to adverse health effects of environmental chemicals. The field of stress measurement primarily relies on individual questionnaire or biomarker data to assess the occurrence of stressful events [18], conditions that might produce stressful experiences [19], recent perceptions of stress [20], or the mental health sequelae of chronic stress (i.e., depression, anxiety).

In contrast, large epidemiological studies that seek to evaluate whether chronic psychosocial stress increases susceptibility to chemical exposures are often unable to assess stress at the individual-level. As a result, they often rely on administrative indicators (e.g., crime, poverty rates) uniformly assessed across heterogeneous communities, as proxy measures to capture the presence of social stressors (e.g., lack of neighborhood safety, financial stress), and, by extension, psychosocial stress. However, because the impact of any stressor depends on an individual’s appraisal (i.e., how one experiences, perceives, or interprets the event), measuring social stressors can result in imprecise assessments of psychosocial stress. Moreover, based on evidence that psychosocial stress levels are high in low SEP areas [21, 22], most epidemiological studies of combined social and environmental effects have primarily used census-derived socioeconomic position (SEP) and demographic measures as a proxy for both a range of social stressors and for psychosocial stress *per se* [14]. Few studies have tested the assumption that SEP indicators are an appropriate proxy. As a result, it remains unclear how well SEP indicators capture exposure to social stressors and psychosocial stress; if these indicators are, in fact, weak proxies, it would limit the interpretability of contextual SEP effects, and hamper identification of possible causal mechanisms. As an alternative approach, some studies aiming to focus on psychosocial stress have examined other single social stressors, choosing stressors that are unlikely to be appraised positively (e.g., exposure to violence [5]). Both approaches suffer from unmeasured confounding insofar as they cannot account for, or distinguish amongst, the constellation of social stressors that can contribute to differential physiological susceptibility to chemical exposures.

Spatial correlation, or common clustering between distinct exposures – and discerning its impact on possible confounding and effect modification – is a key measurement challenge for social-environmental epidemiology. For example, traffic-related air pollution may be inherently confounded by traffic-related noise [23, 24], complicating the interpretability of effects for either exposure. Combining data on multiple social stressors addresses some of the concerns identified above, but a further methodological challenge is that publicly-available indicators are often aggregated to different administrative spatial scales by data source and type (i.e., police precincts, census tracts). Moreover, a number of different stressor indicators for the same construct may be available (e.g., multiple felony crime indicators – assault, robbery, or burglary), and it remains under-explored how well each of these various stressor indicators captures the intended psychosocial construct. As such, using only a single indicator of that construct may or may not be sufficient for capturing spatial distributions in these exposures. Thus, with reproducible geo-statistical methods to elucidate common spatial variation in social stressors and chemical exposures across large cohorts, we will improve our ability to reduce confounding and design studies appropriately powered to disentangle separate and combined effects.

Here, we present a spatial approach for characterizing co-varying social and environmental exposures. To demonstrate this approach, we use refined geographic analyses to examine intra-urban relationships across multiple exposures in New York City (NYC), where social, economic, and physical environmental conditions vary widely. Exposure data are drawn from multiple publicly-available administrative databases to capture dimensions of the social environment, and air pollution data are from the New York City Community Air Survey (NYCCAS). We quantify spatial relationships across this broad set of social stressor indicators, and between these stressor indicators and air pollution. We use geographic information systems (GIS)-based methods to: a) facilitate comparisons across different, incongruent administrative areal units, and b) explore potential effects of areal unit and spatial autocorrelation on observed associations between stressors and air pollution. Finally, we present an exploratory ecologic analysis of spatial confounding and effect modification by social stressors in the relationship between nitrogen dioxide (NO_2_) and childhood asthma exacerbations, to illustrate the risks associated with mis-specification of spatially-patterned exposures and susceptibility.

## Methods

### Data sources and aggregation

#### Outdoor air pollution – NYYCAS

**Figure 1:**
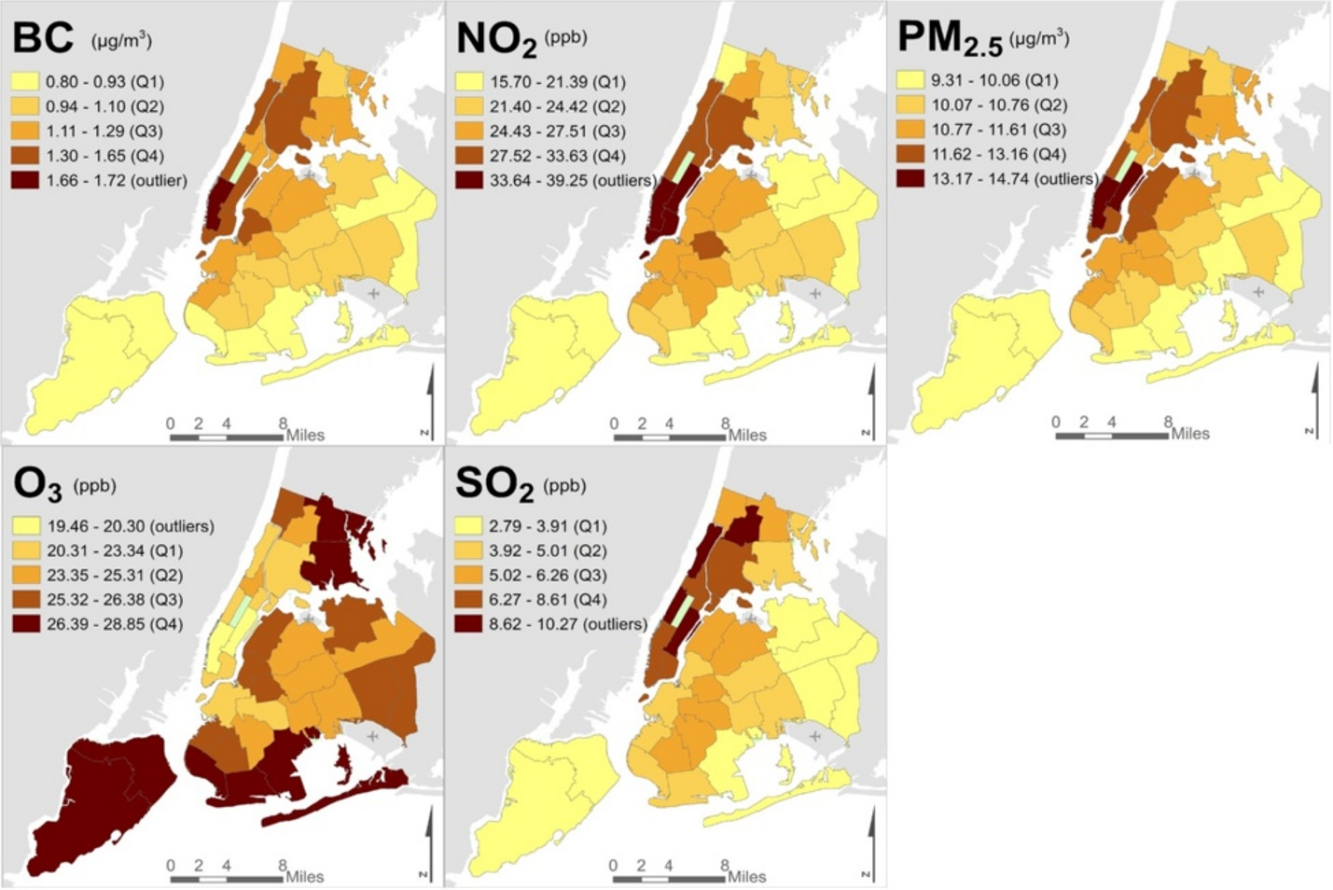
**NYCCAS 2008–2009 mean pollution concentrations, by UHF.**

NYCCAS is a surveillance program of the NYC Department of Health & Mental Hygiene (DOHMH), designed to inform local air quality initiatives. Spatial saturation monitoring was performed year-round across all NYC communities; study design and protocols have been explained in detail elsewhere [25]. Land Use Regression techniques were used to model intra-urban variation in ground-level fine particulate matter (PM_2.5_), black carbon (BC), nitrogen dioxide (NO_2_), wintertime sulfur dioxide (SO_2_), and summertime ozone (O_3_) [26]. Fine-scale pollutant concentration surfaces were averaged to five administrative units (UHF, CD, PP, SD, USCT), for comparability with social stressor indicators (Figure 1).

#### Social stressors – variable selection and formulation

**Figure 2:**
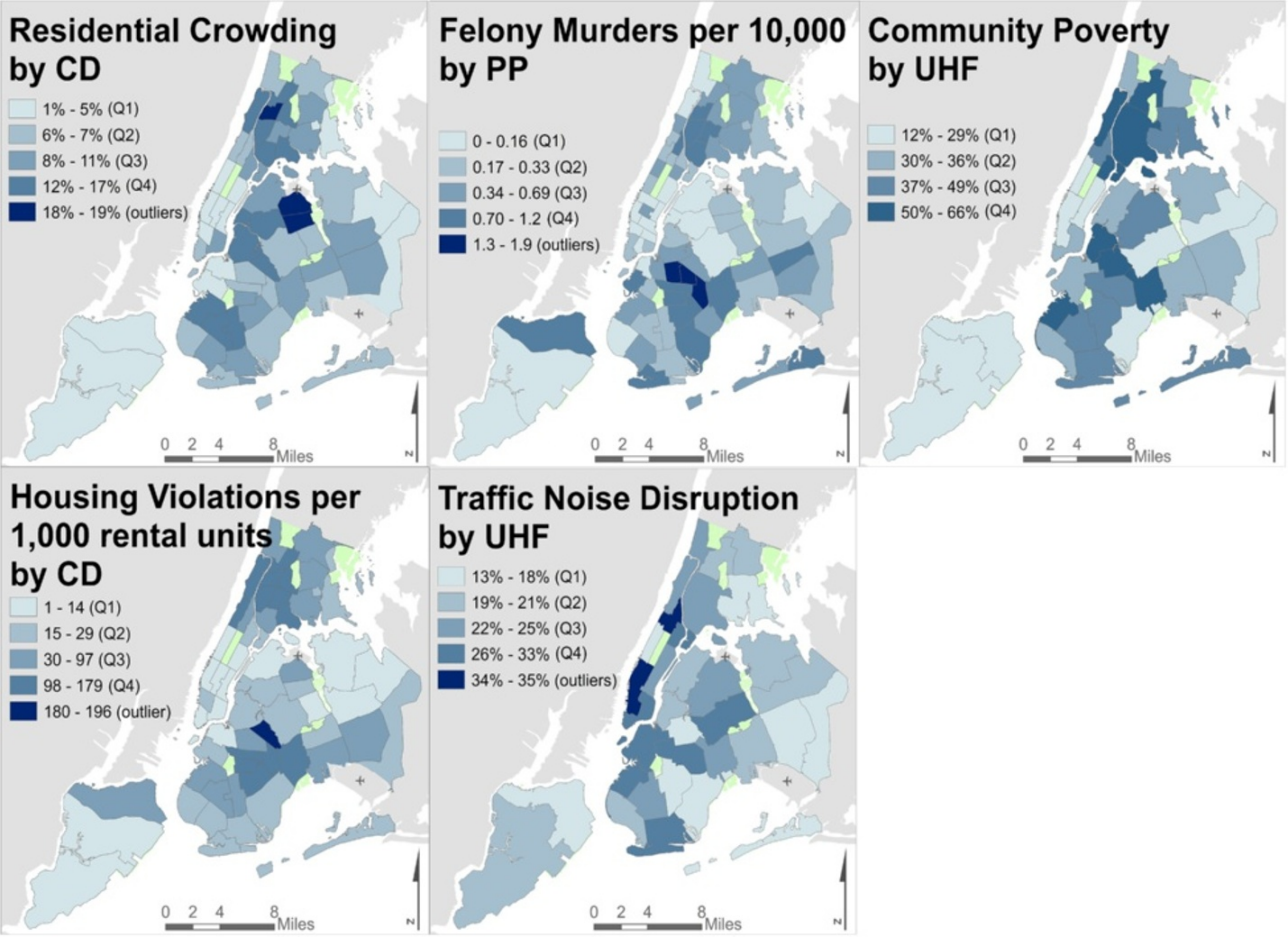
**Social stressor indicators, by differing administrative units.**

We identified 29 administrative indicators that may provide information on exposure to social stressors collected by NYC government agencies and the US Census Bureau (Table 1, Figure 2). Administrative indicators of social stressors were reported at five areal units: Police Precincts (PP) (n =74), Community Districts (CD) (n =59), United Health Fund areas (UHF) (n =34), School Districts (SD) (n =32), and census tracts (USCT) (n =2,111). We obtained multiple indicators of six stressor constructs, to evaluate whether indicators for the same construct follow similar spatial patterns; for example, under ‘physical disorder,’ we explored five different indicators, to enable exploration of both within-construct and between-construct spatial heterogeneity. Administrative indicators were selected to capture key social stressors as identified by focus groups [27] and by prior literature, including: violence and crime [28], neighborhood disorder [19, 29], and noise [30]. Inclusion criteria for the current study required: a) reliable and uniform data quality and interpretability across all communities, b) citywide coverage, and c) approximately concurrent temporality with air pollution data (2008–2010). We included Census-derived area-level SEP and racial composition indicators, to examine how these indicators might co-vary with administrative indicators of social stressors. We excluded indicators with known biases (e.g., differential reporting of and conviction for felony rape [31]) or complicated interpretability with respect to chronic stress (e.g., green space may represent access to recreation, or perceived unsafe areas).

**Figure 3:**
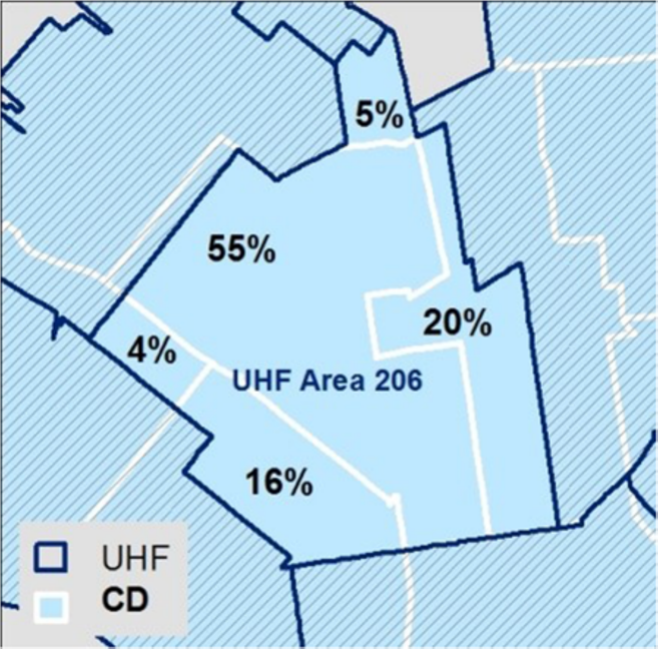
**Areal weighting by proportional coverage.**

To address the challenge of multiple administrative areal units, we applied GIS-based techniques to derive and validate area-weighted prevalence estimates at a common unit of analysis. First, we calculated percent geographic overlap between all administrative units to derive proportional-coverage weights matrices, then reformulated all stressor indicator prevalence to UHF (the reporting unit for hospital admissions and health survey data), to enable correlation analysis across indicators (Figure 3). We chose an area-based technique, rather than population density-based, to maximize interpretability, as we have no evidence that stressor prevalence varies in proportion to population density. We aggregated census data from tracts to UHF areas based on centroid containment, excluding tracts with fewer than 20 residents.

**Table 1:** **Social stressor indicators**

***Stressor construct***	***Administrative indicator***	***NYC agency administrative data source***	***Scale***	***Date***
**Crime & Violence**	Felony Larceny Crimes	NYC Police Department (NYPD)	PP	FY2009
	Felony Murder and non-negligent manslaughter	NYPD	PP	FY2009
	Felonious Assault	NYPD	PP	FY2009
	Felony Robbery	NYPD	PP	FY2009
	Felony Burglary	NYPD	PP	FY2009
	Perceived Lack of Neighborhood Safety [self-report (SR)]	DOHMH Community Health Survey (CHS)	UHF	2010
**Physical Disorder**	Small parks not acceptably clean	NYC Parks Department	CD	FY2009
	Sidewalks not acceptably clean	NYC Mayor’s Office of Operations (MOoO)	CD	FY2009
	Serious housing violations	NYC Dept. of Housing Preservation and Development	CD	2009
	Air Quality complaints	NY State Department of Environmental Protection	CD	FY2009
	Crowding (>1 occupant/room)	US Census American Community Survey (ACS)	USCT	2005-09
**Access to Healthcare**	No insurance coverage (SR)	CHS	UHF	2009
	Went without needed medical care (SR)	CHS	UHF	2009
	Without personal care provider (SR)	CHS	UHF	2009
	Public Health Insurance enrollment	MOoO	CD	FY2009
**Noise disruption**	Frequent noise disruption (3+ times/wk) (SR)	CHS	UHF	2009
	Noise disruption, by neighbors, traffic (SR)	CHS	UHF	2009
**School-related stressors**	Students in schools exceeding capacity	NYC Department of Education (DOE)	SD	2006-07
	School buildings in good to fair condition	DOE	SD	2006-07
	Average daily student attendance	DOE	SD	2006-07
	Substantiated cases of Child Abuse/Neglect	NYC Administration of Child Services	CD	2009
**Socioeconomic Position (SEP)**	Living below 200% Federal Poverty Line	ACS	USCT	2005-09
	Delayed rent or mortgage payment in past year (SR)	CHS	UHF	2009
	Food Stamp program enrollment	MOoO	CD	FY2009
	Less than high school education (SR)	CHS	UHF	2009
	Unemployed <1 year	ACS	USCT	2005-09
	Non-White racial composition	ACS	USCT	2005-09
	African American (Non-Hispanic) racial composition	ACS	USCT	2005-09
	Hispanic ethnic composition	ACS	USCT	2005-09

Because the above areal weighting method cannot account for within-area variation in aggregate data, we introduce a technique quantifying the potential for exposure misclassification due to areal weighting. Using three high-resolution NYCCAS continuous (smooth) pollution surfaces with differing spatial patterns (PM_2.5_, SO_2_, and O_3_), we calculated mean concentrations at multiple administrative units (CD, PP, SD, and UHF). We then applied the same areal weighting method to reformulate concentrations at CD, PP, and SD to UHF units, enabling a comparison of reformulated mean concentrations to the original, ‘known’ area-level concentrations. For this validation, we do not assume that pollution patterns reflect stressor patterns – rather, these three different smooth surfaces (known spatial processes) merely enable analysis of the reproducibility of areal reformulation across administrative units. Using a percent-error tolerance of 5%, and examining similarity in the density distribution (Additional file 1: Figure S1), we found that CD and PP units were reasonably reformulated to UHF for global analysis, but SDs (the largest spatial unit) were not. In sensitivity analyses, we confirmed that detection of autocorrelation was consistent between original units and reformulated values. Calculations were performed in ESRI ArcGIS, v10, and R Statistical Software, v2.11.

### Spatial autocorrelation

We examined potential impacts of spatial autocorrelation – the geographic principle that near areas are more similar than are far areas (and thus non-independent) [32] – on bivariate measures of association between area-level exposures. Autocorrelation structures can be operationalized in statistical models as spatial weights (W_*ij*_), wherein either centroid distance or contiguity (i.e., shared boundaries) is quantified for each observation pair. Given NYC’s irregularly-sized and shaped administrative units, we used first-order (Queen) contiguity, wherein areas sharing *any* boundary are neighbors (W_*ij*_ =1), else non-neighbors (W_*ij*_ =0). We used the Moran’s I statistic to detect non-random spatial clustering in each variable (as summed cross-products of deviations between neighboring units, and deviation from overall mean) [33]. We sensitivity-tested spatial weights using inverse distance between unit centroids.

We then examined potential impacts of spatial autocorrelation in bivariate Simultaneous Autoregressive (SAR) models, which apply spatial weights and Moran’s I to identify model misspecification, potentially due to spatial dependence, in Ordinary Least Squares (OLS) residuals. Where appropriate, we used SAR to derive pseudo-r values [34], which, though not directly comparable to Pearson rho values (i.e., do not represent proportion of variance explained), do effectively rank shared variance across covariates. While most stressors displayed spatial clustering across area units, only 20% of bivariate OLS comparisons revealed residual autocorrelation, calling for SAR. As most (88%) of SAR pseudo-r values did not differ substantially from OLS rho values, we report OLS as the main results here. SAR results and model specification (i.e., spatial error vs. lag models) are reported in Additional file 2: Table S2.

### Statistical analysis

We characterized intra-urban variability and quantified spatial correlations across social stressors, and between stressors and pollution, using Pearson correlation coefficients and SAR pseudo r-values, calculated at the original area unit (for covariates reported at the same administrative unit), else at UHF. To identity suites of spatially co-varying social stressors, we used exploratory factor analysis (EFA) including all stressors aggregated to UHF. We used orthogonal (varimax) rotation, and identified the optimal number of factors using scree plots, covariance eigenvalues, and factor interpretability.

To evaluate whether the factor solution was driven by data density (i.e., number of indicators available within each construct), or covariance due to shared substantive or spatial variance across stressor variables, we employed multiple sensitivity analyses: 1) we separately removed five “redundant” indicators within constructs (rho ≥0.8) to ensure that the factor solution were robust to imbalance in number of indicators by construct, and 2) because some indicators may not solely indicate psychosocial stress pathways (e.g., noise exposure may act through auditory pathways), we separately removed each, then repeated analyses. Sensitivity analysis for autocorrelation impacts on measures of association revealed that our data did not require adjustment for spatial dependence in factor analysis (e.g., [35]). Analyses were performed in ESRI ArcGIS v10, OpenGeoDa v0.9.9.14, and R v2.11.

### Ecologic analysis: social stressors, NO_2_ and child asthma exacerbation

The primary objective of this ecologic analysis is to demonstrate how this spatial approach can be operationalized, and to explore the potential impacts of social stressor indicator selection or spatial mis-specification in stressor patterns, for social-environmental analyses. From the EFA, we identified suites of spatially-correlated stressors (factors) and derived factor scores for each UHF area. Factors were then examined as potential effect modifiers in the relationship between UHF-level mean NO_2_ concentration and asthma Emergency Department (ED) visits rates for children aged 0–14 years during 2008–2010 [from the New York State Department of Health Statewide Planning and Research Cooperative System (SPARCS)]. We used single-predictor and multi-variable SAR models to evaluate the relationship between a cross-sectional ecologic exposure (i.e., NO_2_) and child asthma ED visits by UHF. To examine potential modification of NO_2_ effects by stressor factors, we stratified the 34 UHF areas at the median factor score, and sensitivity-tested models stratifying each factor at a score of 0.

## Results

### Spatial heterogeneity and correlation among social stressors

We identified significant intra-urban variability and spatial autocorrelation within both social stressors and pollutant concentrations (Table 2). Social stressors were not consistently correlated with each other, even within construct (e.g., among indicators intended to capture similar aspects of the social environment) (Table 3). For example, rates of self-reported noise disruption varied by noise source, and noise from traffic and from neighbors were highly uncorrelated (rho =0.01). Likewise, correlations among indicators of area-level SEP varied widely (rho = -0.05 to 0.89). Stressor indicators related to crime and safety were strongly positively correlated, except for those related to property crimes (i.e., larceny, burglary).

**Figure 4:**
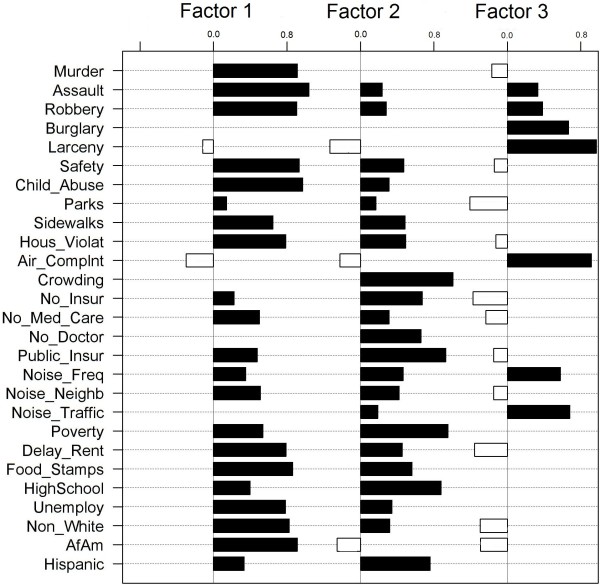
**Factor Analysis 3-factor solution loadings.**

**Figure 5:**
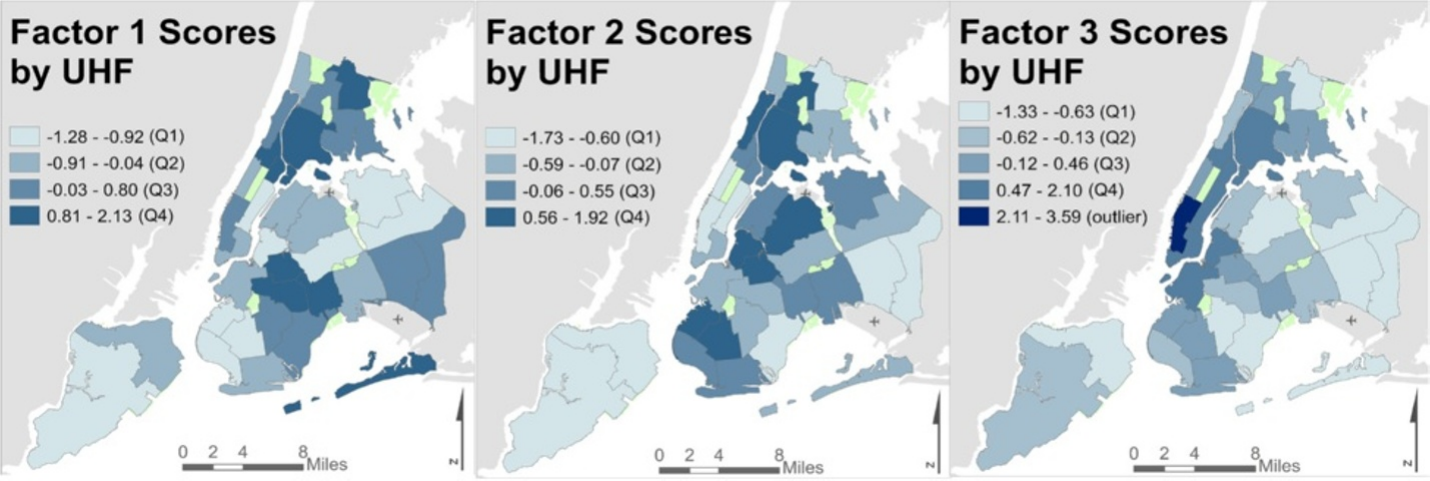
**Social stressor factor scores, by UHF.**

EFA suggested a three-factor solution summarizing the inter-relationships among social stressor indicators (Figure 4). These three factors explained 92.7% of overall spatial variance across 26 social stressor indicators, and each exhibited distinct spatial patterning (Figure 5). Factor 1 (‘violent crime and physical disorder’) included indicators related to violent crime, perceived lack of safety, unclean sidewalks, housing violations, and low area-level SEP (i.e., delayed rent/mortgage payments, Food Stamps enrollment, unemployment, proportion non-white and African American population). Factor 2 (‘crowding and poor access to resources’) included indicators related to residential crowding, poor access to healthcare resources, and other area-level SEP indicators (i.e., low educational attainment, high proportion Hispanic population). Factor 3 (‘noise complaints and property crime’) included indicators related to noise and air pollution complaints, mental health treatment, and property crimes, but not SEP. These factors explained 38%, 35% and 28% of variance, respectively, and were robust to sensitivity analyses.

**Table 2:** **Area-level summary statistics**

Administrative indicator	Mean	Min	Max	SD	Moran’s I ^†^
Felony Larceny Crimes/10,000 persons	50.89	17.01	457.69	57.90	0.38**
Murder/10,000	0.43	0.00	1.85	0.37	0.46**
Felonious Assault/10,000	15.46	1.82	42.34	9.00	0.39**
Felony Robbery/10,000	19.59	3.08	48.68	9.11	0.34**
Felony Burglary/10,000	18.48	5.52	93.70	10.52	0.14*
% Perceived Lack of Neighborhood Safety	30.39	4.70	64.70	16.93	0.25*
% Parks not acceptably clean	20.46	0.00	51.00	11.52	0.34**
% Sidewalks not acceptably clean	3.07	0.20	10.80	2.16	0.52**
Serious housing violations/1,000 Units	53.87	1.40	195.80	51.11	0.57**
Air Quality complaints/10,000	12.50	3.87	56.76	11.89	0.70**
% Crowding	7.95	1.73	16.28	3.67	0.24*
% With no insurance coverage	15.42	2.94	29.62	5.69	0.31*
% Went without needed medical care	11.56	3.58	19.68	3.79	0.20
% Without personal care provider	16.45	8.12	32.36	5.96	0.08
Public Health Insurance enrollment	2801.64	417.10	5356.22	1274.07	0.41**
% Frequent noise disruption	19.86	11.38	35.33	5.81	0.08
% Traffic noise disruption	21.91	12.98	35.21	5.61	0.07
% Neighbor noise disruption	19.63	7.86	30.32	5.28	0.09
% Students in schools exceeding capacity	16.00	0.00	41.70	12.81	0.10
% School buildings in good to fair condition	33.16	1.00	57.00	12.00	0.14
% Average daily student absenteeism	9.94	6.67	14.75	1.86	0.41**
Cases of Child Abuse/Neglect	26.84	2.69	87.82	21.95	0.62**
% Living below 200% federal poverty line (FPL)	37.16	12.15	65.82	13.04	0.32*
% Delayed rent or mortgage payment	15.78	4.99	29.43	6.86	0.25*
Food Stamp program enrollment/10,000	1638.20	186.31	3888.49	1040.26	0.54**
% Less than high school education	13.47	2.80	35.70	8.10	0.10
% Unemployed <1 year	8.38	4.38	14.24	2.44	0.55**
% Non-White racial composition	63.32	20.34	97.98	23.31	0.28*
% African American (Non-Hispanic)	23.31	1.64	72.62	22.54	0.35*
% Hispanic ethnicity composition	26.25	6.33	64.67	16.80	0.53**
**Mean pollution concentration, by UHF**	**Mean**	**Min**	**Max**	**SD**	**Moran's I**
BC (abs)	1.12	0.80	1.72	0.22	0.57**
NO_2_ (ppb)	25.13	15.70	39.25	5.20	0.57**
PM_2.5_ (μg/m^3^)	11.08	9.31	14.74	1.29	0.56**
SO_2_ (ppb)	5.40	2.79	10.27	1.94	0.52**
O_3_ (ppb)	24.85	19.46	28.85	2.19	0.43**

*p <0.01.

**p <0.0001.

^**†**^Moran’s I values near zero indicate random dispersion; positive values indicate spatial autocorrelation.

**Table 3:** **Correlation among indicators of social stressors**

	Larceny	Murder	Assault	Robbery	Burglary	Safety	Child abuse/neglect	Parks unclean	Sidewalks unclean	Serious housing violations	Air quality complaints	Crowding	No insurance	Without needed care	No medical provider	Public health insurance	Freq. noise disrupt	Traffic noise disrupt	Neighbor noise disrupt	Delayed rent/mortgage	Food Stamp enrollment	Less high school education	Unemployed	Poverty	% Non-White	% African American	% Hispanic
Larceny	1																										
Murder	-0.13	1																									
Assault	0.23	**0.68**	1																								
Robbery	0.33	**0.60**	**0.89**	1																							
Burglary	**0.84**	0.13	0.40	0.50	1																						
Safety	-0.33	**0.73**	**0.82**	**0.73**	-0.06	1																					
Child abuse	-0.23	**0.70**	**0.85**	**0.70**	-0.01	**0.85**	1																				
Parks unclean	-0.44	0.20	0.05	0.02	-0.26	0.15	0.07	1																			
Sidewalks unclean	-0.16	**0.74**	**0.64**	0.59	0.41	**0.65**	0.58	0.10	1																		
Housing violations	-0.26	**0.66**	**0.71**	**0.62**	0.08	**0.84**	**0.67**	0.09	0.55	1																	
Air quality complaints	**0.84**	-0.34	-0.05	0.03	0.43	-0.37	-0.36	-0.38	-0.26	-0.36	1																
Crowding	-0.21	0.17	0.25	0.30	0.22	0.39	0.27	0.14	0.48	0.46	-0.20	1															
No insurance	-0.35	0.21	0.21	0.18	-0.22	0.51	0.28	0.30	0.38	0.5	-0.43	**0.63**	1														
Without care	-0.30	0.41	0.41	0.39	0.03	0.53	0.38	0.15	0.35	**0.63**	-0.29	0.34	0.50	1													
No provider	-0.03	0.09	0.22	0.22	0.09	0.44	0.15	-0.05	0.32	0.43	-0.03	**0.60**	**0.63**	0.22	1												
Public HI	-0.44	0.45	0.53	0.50	0.04	**0.70**	**0.74**	0.28	0.54	**0.63**	-0.52	**0.79**	0.56	0.37	0.46	1											
Freq. noise disrupt	0.23	0.11	0.54	0.52	0.13	0.39	0.34	-0.06	0.24	0.30	0.22	0.33	0.23	0.25	0.24	0.39	1										
Traffic noise	0.45	-0.19	0.14	0.18	0.10	-0.02	-0.11	-0.20	-0.01	-0.09	0.45	0.05	-0.03	-0.04	0.17	0.01	**0.73**	1									
Neighbor noise	-0.16	0.50	0.47	0.26	-0.06	0.56	0.53	0.36	0.47	0.51	-0.31	0.41	0.41	0.26	0.18	0.52	0.42	0.01	1								
Delayed rent	-0.37	**0.67**	**0.65**	0.52	**0.07**	**0.82**	**0.65**	0.28	**0.65**	**0.81**	-0.54	0.53	**0.62**	0.56	0.44	**0.67**	0.13	-0.32	**0.64**	1							
Food stamp	-0.27	**0.66**	**0.82**	**0.75**	0.10	**0.84**	**0.92**	0.14	**0.70**	**0.74**	-0.38	0.46	0.36	0.40	0.26	**0.85**	0.42	-0.02	0.52	**0.70**	1						
% < High school	-0.25	0.31	0.49	0.49	0.14	**0.66**	0.52	0.04	0.52	**0.61**	-0.28	**0.83**	0.55	0.49	0.55	**0.80**	0.36	0.04	0.31	**0.63**	**0.62**	1					
% Unemployed	0.20	0.39	0.52	0.48	0.3	**0.77**	0.36	0	0.35	0.42	0.04	0.28	0.41	0.46	0.44	0.21	0.40	0.08	0.33	0.42	0.31	0.46	1				
% Poverty	-0.08	0.51	**0.64**	**0.60**	-0.03	**0.75**	**0.78**	0.19	**0.65**	**0.75**	-0.40	**0.79**	0.57	0.42	0.52	**0.92**	0.57	0.18	0.55	**0.63**	**0.89**	**0.80**	0.28	1			
% Non-White	-0.34	**0.64**	**0.62**	0.58	-0.01	**0.79**	**0.71**	0.10	0.50	**0.72**	-0.52	0.37	0.50	0.59	0.44	**0.70**	0.20	0.14	0.30	**0.77**	**0.73**	**0.60**	0.36	0.55	1		
% African American	-0.21	**0.72**	0.55	0.52	-0.02	0.59	0.48	0.15	0.51	0.49	-0.35	0.08	0.17	0.48	0.02	0.24	0	0.25	0.30	**0.63**	0.49	0.11	0.32	0.16	**0.70**	1	
% Hispanic	-0.25	0.14	0.40	0.35	-0.10	0.59	**0.63**	0.01	0.27	**0.63**	-0.32	**0.60**	0.49	0.39	0.55	**0.70**	0.34	0.06	0.23	0.47	**0.64**	0.76	0.20	**0.61**	0.57	-0.06	1

Bold values indicate Pearson rho ≥ 0.60.

To examine whether the geographically distinct patterns of social stressors represented by the three-factor solution provide different, or more comprehensive, information about the distribution of stressor exposures than simply considering any single indicator of area-level SEP, we assessed two commonly-used SEP indicators – area-level poverty (% households below 200% FPL) and low educational attainment (% adults with less than High School education) – across communities in the highest quartile for each of the three stressor factors. Among UHF areas with scores in the highest quartile for Factor 1, the mean poverty rate was 50%; for Factor 2, 53%; and for Factor 3, 41%; compared to the city-wide mean of 37%. Similarly, across UHFs with factor scores in the highest quartile, % less than High School education was above the city-wide mean (13%) for all factors (19%, 24%, and 15%, respectively).

### Social stressors and air pollution

UHF-average concentrations of BC, NO_2_, PM_2.5_, and SO_2_ were all positively correlated (rho = 0.74 to 0.96), and each inversely correlated with O_3_ (rho = -0.69 to -0.90). We identified strong spatial correlations with pollutants [BC, NO_2_, PM_2.5_, and O_3_ (inverse)] only for Factor 3 (‘noise complaints and property crime’) (rho >0.70) (Table 4). Factors 1 and 2 were not correlated with air pollution (rho = -0.07 to 0.08, and 0.04 to 0.12, respectively). Nor were poverty or educational attainment rates highly correlated with pollutant concentrations (rho = 0.01 to 0.17, and 0.01 to 0.11, respectively).

**Table 4:** **Spatial correlation (Pearson rho) between stressor factors and outdoor air pollution, by UHF (n =34)**

	BC	NO _2_	PM _2.5_	SO _2_	O _3_
**Factor 1**	-0.02	-0.01	-0.07	0.08	-0.01
(violent crime and physical disorder)					
**Factor 2**	0.12	0.04	0.06	0.11	0.08
(crowding and poor access to resources)					
**Factor 3**	0.80**	0.83**	0.83**	0.44*	-0.74**
(noise complaints and property crime)					

*indicates statistical significance at p < 0.01, **p <0.0001.

### Ecologic analysis: stressor factors and NO_2_ on childhood asthma ED visit rates

Citywide, during 2008–2010, the mean UHF-level rate of child (0–14 years old) asthma-related ED visits was 6.8%. Mean annual NO_2_ concentrations across UHF areas ranged from 15.7 to 39.3 ppb (mean 25.1 ppb). In separate ecologic regression models for each stressor factor and NO_2_, on asthma ED visits, we found a significant association only for Factor 1 (‘violent crime and physical disorder’); an interquartile range (IQR) increase in Factor 1 was associated with a 3.9% increase in childhood ED visits (p <0.0001). No associations were evident for other factors, or for area-average NO_2_. The association for Factor 1 remained after adjusting for Factors 2 and 3, and NO_2_.

**Figure 6:**
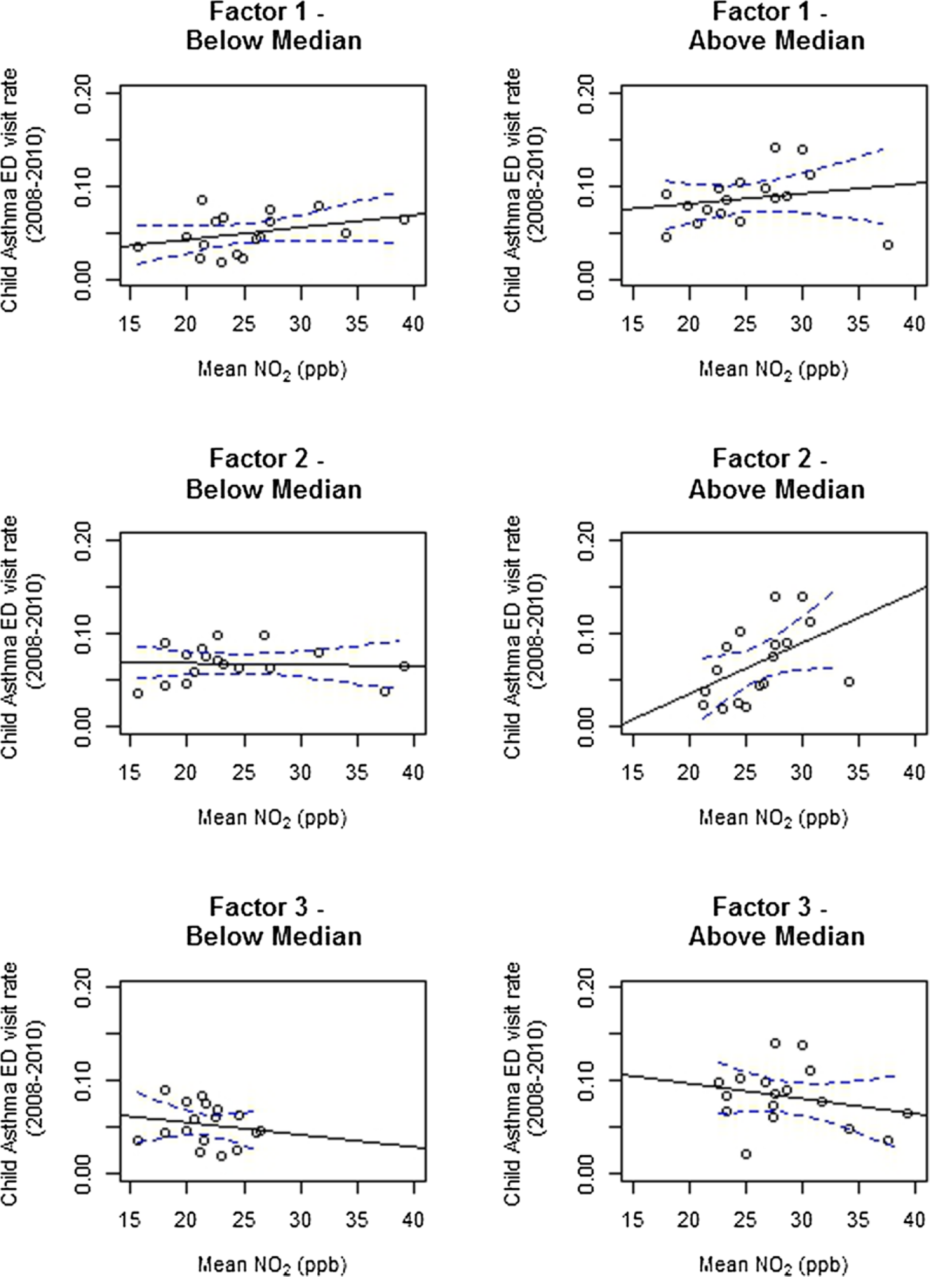
**Modification of the association between area-level NO**_**2**_ **and child asthma ED visit rates by social stressor factors.**

We examined effect modification in the NO_2_-asthma exacerbation relationship by stressor factors (Figure 6), and found significant (p <0.05) modification only by Factor 2 (‘crowding and poor access to resources’); among UHF areas scoring above the median on Factor 2, each 10 ppb increase in area-average NO_2_ was associated with a 5.5% increase in child asthma ED visit rates. Given potential outcome bias for Factor 2 (which included access to health care indicators), we sensitivity-tested this effect using single health care access indicators, finding no significant modification.

We compared these model results to those using area-level poverty rates (Table 1) as the modifier. The association between poverty rates and ED visits was slightly weaker than the association observed for Factor 1 – an IQR increase in % households below 200% FPL conferred a 2.3% increase in ED visits – with a substantially weaker model fit (R^2^ = 0.24 vs. 0.54). We found no modification of the association between NO_2_ and asthma ED visits by area-level poverty rates.

### Additional sensitivity analyses

To evaluate the sensitivity of correlations among stressor indicators to the unit of aggregation (Modifiable Areal Unit Problem (MAUP) [36]), we aggregated two high-resolution spatial data sets (NYCCAS smooth surface air pollutants, and census tract variables) to each administrative unit. Correlations were consistent across units, supporting the reliability of our findings (Additional file 2: Table S1). We also tested the sensitivity of autocorrelation detection to the spatial weighting method (i.e., first-order contiguous neighbors versus inverse distance between area centroids) and unit of aggregation, which did not influence results.

## Discussion

We used GIS-based techniques to quantify relationships across social stressor indicators, and between these potential social stressors and air pollutants across NYC. Our findings call attention to complex spatial patterning across diverse social stressor and SEP indicators, and emphasize the importance of refined social exposure assessment for environmental health research. This spatial approach enables the disentangling of potentially correlated, yet conceptually distinct, chemical and non-chemical exposures – towards better quantifying spatial confounding and effect modification in social-environmental epidemiology.

Importantly, we found that a diverse set of social stressors across NYC are: 1) not consistently correlated, even among indicators that appear to be measuring similar aspects of the social environment (e.g., crime indicators), 2) not consistently correlated with area-level SEP, and 3) not consistently correlated with air pollution. The complexity of relationships among stressor indicators was borne out in factor analysis, which identified three spatially-distinct suites of stressors – ‘violent crime and physical disorder’, ‘crowding and poor access to resources’, and ‘noise complaints and property crime’ – suggesting that co-variation might be driven more by common spatial patterning than by shared meaning. Importantly, these three spatial factors did not represent different levels of socioeconomic position; areas that were similar with respect to area-level SEP did not necessarily have similar prevalence and combinations of other social stressors. As such, using any single stressor (including SEP) measure to serve as a proxy for psychosocial stress may be misleading; because areas that may be similar with respect to area-level SEP measures may differ regarding social stressors, single measures may inadvertently lead to confounding, and fail to capture important nuances of the social environment. It is also worth noting that some communities had high factor scores for more than one stressor factor, underscoring the potential for cumulative effects of multiple exposures in those communities. While the spatial patterning empirically summarized by stressor factors would likely differ between cities and regions, this reproducible approach may be helpful in developing locally appropriate composite social stressor measures.

Leveraging common spatial patterns among social stressors across communities enabled a more comprehensive characterization of social exposures, and perhaps psychosocial stress, and potential interactions with air pollution, which may contribute to social disparities in health. For example, in our ecologic analysis, air pollution was strongly correlated only with the spatial factor corresponding to ‘noise complaints and property crime’ (Factor 3), not with the other factors, or with indicators of area-level SEP. This is noteworthy, as communities with relatively high SEP and better healthcare access loaded relatively strongly on Factor 3 – a result which counters the common assumptions that air pollution would be highest in low-SEP communities, leading to positive confounding in air pollution epidemiology. It is also of note that our only indicator of perceived pollution – air quality complaint rates – loaded strongly on Factor 3, suggesting correlation between spatial patterns in modeled pollution concentrations and perceived air poor quality. The ability of pollution (or its sources) to act as both a chemical and non-chemical stressor is increasingly recognized as an important source of confounding [14].

In our ecologic analysis, we illustrated how modification in the NO_2_-asthma exacerbation association may vary substantively by the selection of social stressors – represented here by our three stressor factors. Conceptually, this ecologic analysis underscores the need for thoughtful selection of stressor indicators, as mis-specification of stressors, which are hypothesized to impart physiologic susceptibility, can substantially alter observed effect modification. Further, empirically grouping social stressors according to spatial relationships may better capture potential physiologic susceptibility patterns, relative to using a single stressor indicator – an observation which is reinforced by our result that area-level SEP indicators did not strongly correlate with stressor factors, and thus are likely inadequate proxies for stressor exposures and psychosocial stress.

Though there are few examples in environmental epidemiology for refined social exposure assessment, our findings recall notions of “unpatterned inequality” in urban resource distribution, wherein communities may be favored in the allocation of some resources, while deprived in others [37]. In a recent study of individual- and area-level associations between SEP and air pollution, Hajat et al. [38] identified regional and intra-urban heterogeneity in the strength and direction of associations between area-level SEP and air pollution using spatially-informed regression models, wherein area-level SEP was positively associated with PM_2.5_ and NO_X_ exposures across a geographic subset of NYC communities. More work is necessary, however, to replicate and refine salient social stressor measures, especially for large geographic cohorts wherein individual-level survey assessments of stress experience (e.g., [12]) or on-foot built environment assessments (e.g., [39]) are generally infeasible.

An alternative explanation for our empirically-derived findings include spurious associations due to unit of analysis (i.e., administrative areas are highly imperfect proxies for communities), measurement error in administrative data, or construct misspecification. Generally, larger administrative areas yield less precise metrics [40]; thus, while we aimed to include the widest variety of administrative indicators of social stressors possible, each indicator was examined at the finest resolution available, and areal units were robust to MAUP effects. Likewise, some stressor indicators may capture aspects of both chemical and non-chemical exposure constructs. For example, some physical disorder indicators are linked with poor mental health, but also with allergen and chemical exposures (e.g., cockroaches, pesticides) – both implicated in asthma etiology. Here, we attempted to minimize such confounding by focusing on stressors hypothesized to act predominantly through psychosocial stress pathways. These interpretation challenges are not, however, unique to this analysis, as administrative indicators are widely employed in social and environmental epidemiology. As such, mixed qualitative and quantitative methods for identifying salient stressors across spatially heterogeneous domains, and for validating administrative indicators against community- and individual-level stress experience (e.g., [41]), are important for improving reliability of administrative indicators for environmental epidemiology.

We aimed to develop and validate broadly applicable methods for quantifying common spatial patterning across urban chemical and non-chemical exposures. The NYCCAS fine-scale air pollution data enabled examination of spatial correlations across pollutants – and between pollution and social stressors – and provided fine-scale surfaces for validation of areal re-aggregations. GIS-based sensitivity analyses lend confidence to our quantitative findings. First, our validation method for areal weighting of incongruent spatial units could utilize any smooth surface supplying a known underlying distribution (e.g., elevation raster, kernel density surface), ideally with a scale of variability similar to (or more refined than) the re-aggregated exposure of interest. Though areal reformulation may induce local exposure misclassification, due to unknown within-area variability, our approach is useful for exploring global spatial confounding patterns. Second, sensitivity testing for MAUP effects and autocorrelation improved our understanding of spatial correlations, providing insights for future spatially-informed multi-variable modeling of social-environmental interactions.

## Conclusions

In conclusion, our city-wide examination of social stressors and air pollution in one U.S. city highlight the utility of spatial analysis for disentangling the separate and combined effects of chemical and non-chemical exposures. The process presented for systematically identifying and assimilating area-based administrative indicators of social stressors, and deriving empirical spatially-covariant composites can minimize of confounding among social stressors, and between social stressors and air pollution. Our findings demonstrate that selection of social stressors and geographic scale may substantially alter observed effect modification, caution against using single SEP indicators as proxies for social stressors, and demonstrate the risks associated with mis-specification of social stressor exposures. Empirical studies with stronger validated and spatially-informed measures of social stressor exposures are needed to better understand spatial confounding and joint effects between chemical and non-chemical stressors.

## Electronic supplementary material

Additional file 1: **Kernel density plots comparing pollutant (wintertime PM**_**2.5**_ **and SO**_**2**_ **, and summer O**_**3**_ **) distributions, by UHF (row 1) and areas reformulated to UHF from other administrative units (rows 2-4).** (JPEG 117 KB)

Additional file 2: **Supplemental materials – Areal reformulation and Spatial Regression.** (DOCX 36 KB)

## Abbreviations

ACS: United States Census Bureau American Community Survey
BC: Black carbon
CD: Community district
CHS: Community health survey
DOHMH: NYC Department of Health & Mental Hygiene
ED: Emergency Department
EFA: Exploratory factor analysis
GIS: Geographic information systems
IQR: Interquartile range
MAUP: Modifiable areal unit problem
NO_2_: Nitrogen dioxide
NYC: New York City
NYCCAS: New York City Community Air Survey
O_3_: Ground-level ozone
OLS: Ordinary least squares
PM_2.5_: Fine particulate matter <2.5 micrograms per cubic meter
PP: Police precinct
SAR: Spatial (Simultaneous) autoregressive
SD: School District
SEP: Socioeconomic position
SO_2_: Sulfur dioxide
UHF: United Health Fund Area
USCT: United States Census Tract.
