# Supplementary figures and images for: Social stressors and air pollution across New York City communities: a spatial approach for assessing correlations among multiple exposures
Source: Environ Health. 2014 Nov 6;13:91. doi: 10.1186/1476-069X-13-91 (PMC4240877; doi:10.1186/1476-069X-13-91)

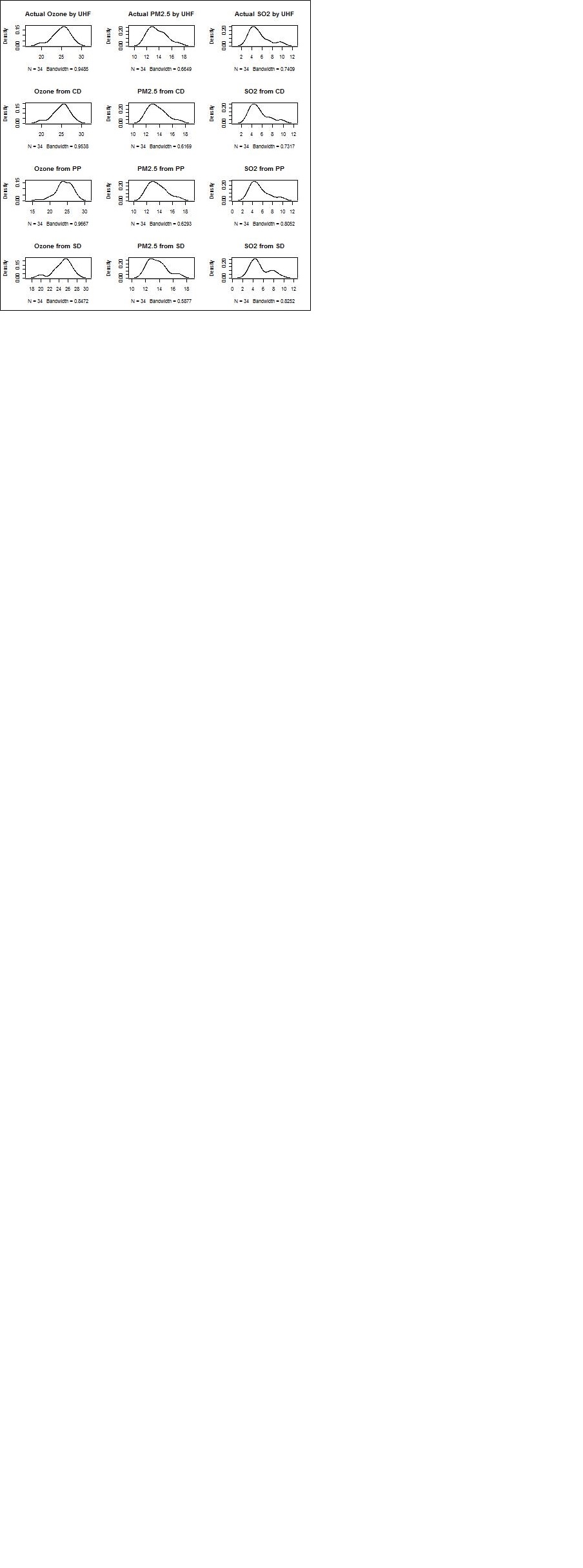

Supplement: Supplementary file 1 — Additional file 1: Kernel density plots comparing pollutant (wintertime PM 2.5 and SO 2 , and summer O 3 ) distributions, by UHF (row 1) and areas reformulated to UHF from other administrative units (rows 2-4). (JPEG 117 KB) [file 12940_2014_796_MOESM1_ESM.jpeg]
