# Supplementary material for: Social stressors and air pollution across New York City communities: a spatial approach for assessing correlations among multiple exposures
Source: Environ Health. 2014 Nov 6;13:91. doi: 10.1186/1476-069X-13-91 (PMC4240877; doi:10.1186/1476-069X-13-91)
Supplement: Supplementary file 2 — Additional file 2: Supplemental materials – Areal reformulation and Spatial Regression. (DOCX 36 KB) [file 12940_2014_796_MOESM2_ESM.docx]

**SUPPLEMENTAL MATERIALS**

**Areal weighting method validation**

To validate our areal weighting approach to reformulating incongruent administrative indicators, we aggregated three high-resolution air pollution surfaces with differing spatial patterns to each administrative unit, and then reformulated CD-, PP- and SD-level concentrations to UHFs. Comparing the *actual* versus *reformulated* UHF-level concentrations across pollutants and administrative units indicates how well this method can re-produce global spatial variation, where underlying within-area variability is unavailable. Density distribution plots and bandwidth (Figure S1) validated CD and PP for reformulation to UHFs, but not SDs.

**Spatial Regression**

We explored multiple spatial statistical techniques accounting for spatial dependence in bivariate correlations, including Geographically Weighted Regression (GWR), Conditional Autoregressive (CAR), and selected Spatial (Simultaneous) Autoregressive (SAR) model as most appropriate to NYC administrative data, given irregular unit shape and size. Geographically Weighted Regression (GWR) allows regression coefficients to vary across space (i.e., non-stationarity); each observation (e.g., sampling point or areal unit) is the target of a separate regression spatially weighted against the entire domain [1]. GWR has greatest utility in multivariate models for which an inverse-distance weighting scheme is desirable (e.g., proximity analysis), and for research questions focusing on *locally-varying* predictor-outcome relationships. Conditional Autoregressive (CAR) and Spatial (Simultaneous) Autoregressive (SAR) models account for spatial autocorrelation *globally*, but CARs specify a symmetric covariance matrix. As such, spatial weights for CAR often include continuous inverse distance decay, not ideal for irregularly-shaped and -sized areal units [2,3]. Some analyses have reported negligible differences between SAR and CAR results [4,5], but SAR requirements (e.g., flexible spatial weights definition, non-symmetric covariance) better match NYC administrative data.

SAR specification begins with diagnostic tests for spatial autocorrelation (e.g., Moran’s I p < 0.05) on single-predictor Ordinary Least Squares (OLS) regression residuals. Autocorrelation among area-level measures may be caused either by: 1) underlying social and/or chemical processes leading to inherent spatial clustering (e.g., higher air pollution concentrations closer to fixed sources), or 2) through “spill-over effects,” a mismatch between the true scale of the underlying process and the administrative unit used (e.g., a neighborhood split across two Police Precincts).

To account for residual autocorrelation, SAR incorporates a *lag* (SAR*_lag_*) or *error* (SAR*_err_*) term [6,7]. Generally, where residual autocorrelation is inherent in the predictor variable(s), SAR*_lag_* models may be more appropriate, applying a weighted autoregressive term (*Wy*) to the response variable (*y* = *ρWy* +*xβ* + *ɛ, with ɛ =* is a vector of *iid* error terms). SAR*_err_* models are useful when spatial dependence is observed primarily in residuals, and incorporate an autoregressive error term (*y* = *xβ* + *ɛ*, with *ɛ* = *ʎW ɛ* + *u*, a vector of spatially correlated error terms, and *u* is a vector of *iid* errors) [8,9]. Having no *a priori* hypothesis about the nature of spatial processes operating across our multiple indicators, we assumed that different units and variables might give rise to diverse autocorrelation structures. As such, we referred to Lagrange Multiplier test statistics to specify SAR model-type (e.g., error or lag), following standard decision-making criteria [7, pp. 196-200].

Despite widespread univariate autocorrelation, relatively few (20%) bivariate comparisons called for SAR. As such, the main analysis prioritized comparability among r-values, and referred only to OLS r-values to estimate spatial correlation. Model fit was improved in all SAR, measured by Log Likelihood Ratio test [7]. SAR model-type was not patterned by administrative unit, or by stressor construct; 88% (n = 63) of comparisons called for an *error* model, versus a *lag* model (n = 9). Figure S2 shows SAR pseudo-r-values, and illustrates the irregular nature of spatial dependence structures across units of aggregation and constructs. Though not directly comparable, all SAR pseudo-r-values were stronger than OLS r-values. The magnitude of that increase, however, varied substantially: among SAR*_err_*, the mean difference was 0.34 (range 0.02 to 0.81), and among SAR*_lag_*, the mean difference was on average less (mean=0.14, range 0.01 to 0.42), but also highly variable.

Importantly, SAR specification is determined jointly by all model covariates, and thus, multivariate epidemiological model specification depends on the underlying structures and spatial interactions present. The predominance of *error*, over *lag*, SAR models in this analysis may be a point of departure for spatial adjustment in complex multi-variable models moving forward. Spatial regression models are relatively new to environmental health research, and incorporating sensitivity tests for spatial autocorrelation in preliminary exploration and variable selection proved beneficial toward understanding SAR model specification.

**References**

1. Fotheringham AS, Brunson C, Charlton ME: *Geographically Weighted Regression: The Analysis of Spatially Varying Relationships*. Chichester: Wiley; 2002.

2. Goovaerts P: **Geostatistical Analysis of County-level Lung Cancer Mortality Rates in the Southeastern United States**. *Geogr Anal* 2010, **42**:32-52.

3. Kelsall J and Wakefield J: **Modeling Spatial Variation in Disease Risk: A Geostatistical Approach.** *J Am Statistical Association* 2002, **97**:692-701.

4. Wall MM: **A close look at the spatial structure implied by the CAR and SAR models.** *Journal of Statistical Planning and Inference* 2004, **121**:311-324.

5. Lichstein JW, Simons TR, Shriner SA, Farnzreb KE: **Spatial Autocorrelation and Autoregressive Models in Ecology.** *Ecological Monographs* 2002, **72**:445-463.

6. Anselin L and Bera AK: **Spatial Dependence in Linear Regression Models with and Introduction to Spatial Econometrics.** In *Handbook of applied economic statistics*. Edited by Ullah A and Giles DEA. New York: Marcel Dekker, Inc; 1998.

7. Anselin L: *Exploring Spatial Data with GeoDa: A Workbook.* Urbana-Champaign, IL: Center for Spatially Integrated Social Science, University of Illinois; 2005.

8. Kissing DW and Carl G: **Spatial autocorrelation and the selection of simultaneous autoregressive models.** *Global Ecol Biogeogr* 2008, **17**:59-71.

9. de Smith M, Goodchild M, Longley P: **Spatial autoregressive and Bayesian modeling.** In *Geospatial Analysis - 4^th^ edition*. [http://www.spatialanalysisonline.com/HTML/index.html]

**Table S1:** Unit of aggregation (MAUP) effects on correlation measures. Correlation coefficients (Pearson r-values) between multiple pollutants and Census variables, aggregated to three different administrative units.

|  | **CD (n=59)** | | | **PP (n=74)** | | | **UHF (n=34)** | | |
| --- | --- | --- | --- | --- | --- | --- | --- | --- | --- |
|  | **% < 200% FPL** | **% Unemp** | **% Non-White** | **% < 200% FPL** | **% Unemp** | **% Non-White** | **% < 200% FPL** | **% Unemp** | **% Non-White** |
| **PM_2.5_** | 0.01 | 0.04 | -0.16 | -0.19 | -0.05 | -0.27 | 0.08 | -0.02 | -0.21 |
| **EC** | 0.13 | 0.17 | -0.01 | -0.11 | 0.01 | -0.20 | 0.14 | 0.07 | -0.10 |
| **NO_2_** | 0.02 | 0.02 | -0.11 | -0.18 | -0.08 | -0.23 | 0.10 | 0.00 | -0.14 |
| **SO_2_** | 0.24 | 0.33 | 0.10 | 0.18 | 0.25 | 0.03 | 0.20 | 0.29 | 0.03 |
| **O_3_** | -0.02 | -0.09 | 0.01 | 0.32 | 0.25 | 0.32 | -0.01 | -0.02 | 0.07 |

**Table S2.** SAR and OLS r-values, by stressor construct. Shaded areas indicate SAR pseudo-r-values (tan = SAR error model, blue = SAR lag model). Bold values indicate rho ≥ 0.60.

[Next page]

| Larceny | 1 |  |  |  |  |  |  |  |  |  |  |  |  |  |  |  |  |  |  |  |  |  |  |  |  |  |  |
| --- | --- | --- | --- | --- | --- | --- | --- | --- | --- | --- | --- | --- | --- | --- | --- | --- | --- | --- | --- | --- | --- | --- | --- | --- | --- | --- | --- |
| Murder | -0.13 | 1 |  |  |  |  |  |  |  |  |  |  |  |  |  |  |  |  |  |  |  |  |  |  |  |  |  |
| Assault | **0.64** | **0.68** | 1 |  |  |  |  |  |  |  |  |  |  |  |  |  |  |  |  |  |  |  |  |  |  |  |  |
| Robbery | 0.33 | **0.60** | **0.90** | 1 |  |  |  |  |  |  |  |  |  |  |  |  |  |  |  |  |  |  |  |  |  |  |  |
| Burglary | **0.90** | 0.13 | 0.40 | **0.62** | 1 |  |  |  |  |  |  |  |  |  |  |  |  |  |  |  |  |  |  |  |  |  |  |
| Safety | -0.33 | **0.73** | **0.82** | **0.73** | -0.06 | 1 |  |  |  |  |  |  |  |  |  |  |  |  |  |  |  |  |  |  |  |  |  |
| Child abuse | -0.02 | **0.85** | **0.88** | **0.82** | -0.01 | **0.85** | 1 |  |  |  |  |  |  |  |  |  |  |  |  |  |  |  |  |  |  |  |  |
| Parks unclean | -0.37 | 0.20 | -0.38 | 0.02 | -0.26 | 0.15 | **-0.74** | 1 |  |  |  |  |  |  |  |  |  |  |  |  |  |  |  |  |  |  |  |
| Sidewalks unclean | -0.16 | **0.83** | **0.86** | **0.81** | 0.41 | **0.76** | **0.86** | 0.10 | 1 |  |  |  |  |  |  |  |  |  |  |  |  |  |  |  |  |  |  |
| Housing Violations | -0.26 | **0.66** | **0.71** | **0.62** | 0.08 | **0.84** | **0.67** | 0.09 | **0.74** | 1 |  |  |  |  |  |  |  |  |  |  |  |  |  |  |  |  |  |
| Air quality complaints | **0.84** | -0.34 | -0.40 | 0.03 | 0.43 | -0.52 | **-0.77** | **-0.80** | -0.26 | -0.36 | 1 |  |  |  |  |  |  |  |  |  |  |  |  |  |  |  |  |
| Crowding | -0.21 | 0.17 | 0.45 | 0.46 | 0.41 | 0.39 | 0.27 | 0.14 | 0.48 | 0.58 | -0.20 | 1 |  |  |  |  |  |  |  |  |  |  |  |  |  |  |  |
| No insurance | -0.44 | 0.21 | 0.41 | 0.18 | -0.41 | 0.51 | 0.28 | 0.30 | 0.38 | 0.50 | -0.43 | **0.63** | 1 |  |  |  |  |  |  |  |  |  |  |  |  |  |  |
| Without care | -0.30 | 0.41 | 0.41 | 0.39 | 0.03 | 0.53 | 0.38 | 0.15 | 0.35 | **0.63** | -0.29 | 0.34 | 0.50 | 1 |  |  |  |  |  |  |  |  |  |  |  |  |  |
| No provider | -0.03 | 0.09 | 0.22 | 0.22 | 0.09 | 0.44 | 0.15 | -0.05 | 0.32 | 0.43 | -0.03 | **0.60** | **0.72** | 0.22 | 1 |  |  |  |  |  |  |  |  |  |  |  |  |
| Public HI | -0.44 | 0.45 | **0.68** | 0.50 | 0.04 | **0.70** | **0.74** | 0.28 | **0.69** | **0.63** | -0.52 | **0.79** | 0.56 | 0.37 | 0.46 | 1 |  |  |  |  |  |  |  |  |  |  |  |
| Freq. noise disrupt | 0.23 | 0.11 | 0.54 | 0.52 | 0.13 | 0.39 | 0.34 | -0.06 | 0.24 | 0.30 | 0.22 | 0.33 | 0.23 | 0.25 | 0.24 | 0.39 | 1 |  |  |  |  |  |  |  |  |  |  |
| Traffic noise disrupt | 0.45 | -0.19 | 0.14 | 0.18 | 0.10 | -0.02 | -0.11 | -0.20 | -0.01 | -0.09 | 0.45 | 0.05 | -0.03 | -0.04 | 0.17 | 0.01 | 0.73 | 1 |  |  |  |  |  |  |  |  |  |
| Neighbor noise disrupt | -0.28 | 0.50 | 0.47 | 0.26 | -0.06 | 0.56 | 0.53 | 0.36 | 0.47 | 0.51 | -0.31 | 0.41 | 0.41 | 0.26 | 0.18 | 0.52 | 0.42 | 0.01 | 1 |  |  |  |  |  |  |  |  |
| Delayed rent | -0.37 | **0.67** | **0.74** | **0.60** | 0.38 | **0.82** | **0.73** | 0.52 | 0.28 | 0.81 | -0.54 | 0.53 | **0.62** | 0.56 | **0.60** | **0.67** | 0.41 | -0.32 | **0.64** | 1 |  |  |  |  |  |  |  |
| Food Stamp enroll | -0.27 | **0.66** | **0.82** | **0.85** | 0.10 | **0.84** | **0.95** | **0.69** | **0.87** | **0.74** | -0.38 | 0.46 | 0.36 | 0.40 | 0.26 | **0.88** | 0.42 | -0.02 | 0.52 | **0.70** | 1 |  |  |  |  |  |  |
| % Less high school | -0.25 | 0.31 | 0.49 | 0.49 | 0.14 | **0.66** | 0.52 | 0.04 | 0.52 | **0.61** | -0.28 | **0.83** | 0.55 | 0.49 | 0.55 | **0.80** | 0.36 | 0.04 | 0.31 | **0.63** | **0.62** | 1 |  |  |  |  |  |
| % Unemployed | 0.20 | 0.39 | 0.52 | 0.48 | 0.3 | **0.83** | 0.36 | 0 | 0.35 | 0.42 | 0.04 | 0.28 | 0.41 | 0.46 | **0.73** | 0.21 | 0.40 | 0.08 | 0.33 | **0.63** | 0.31 | 0.46 | 1 |  |  |  |  |
| % Poverty | -0.08 | 0.51 | **0.72** | **0.60** | -0.03 | **0.75** | **0.78** | 0.19 | **0.80** | **0.80** | -0.40 | **0.79** | 0.57 | 0.42 | 0.52 | **0.92** | 0.57 | 0.18 | 0.55 | 0.63 | 0.91 | 0.80 | 0.28 | 1 |  |  |  |
| % Non-White | -0.34 | **0.64** | **0.72** | 0.58 | -0.01 | **0.79** | **0.71** | 0.10 | 0.50 | **0.72** | -0.52 | 0.37 | 0.50 | 0.59 | 0.44 | **0.70** | 0.20 | 0.14 | 0.30 | **0.77** | **0.73** | **0.60** | 0.36 | 0.55 | 1 |  |  |
| % African American | -0.21 | **0.75** | 0.55 | 0.52 | -0.02 | 0.59 | 0.48 | 0.15 | 0.51 | 0.49 | -0.35 | 0.08 | 0.17 | 0.48 | 0.02 | 0.24 | 0 | 0.25 | 0.30 | **0.68** | 0.49 | 0.11 | 0.32 | 0.16 | **0.70** | 1 |  |
| % Hispanic | -0.25 | 0.14 | 0.40 | 0.35 | -0.10 | 0.59 | **0.63** | 0.01 | 0.27 | **0.63** | -0.32 | **0.60** | 0.49 | 0.39 | 0.55 | **0.70** | 0.34 | 0.06 | 0.24 | 0.47 | **0.64** | 0.76 | 0.20 | **0.63** | 0.57 | -0.06 | 1 |
|  | Larceny | Murder | Assault | Robbery | Burglary | Safety | Child abuse | Parks unclean | Sidewalks unclean | Serious housing violations | Air complaints | Crowding | No insurance | Without needed care | No medical provider | Public health insurance | Freq. noise disrupt | Traffic noise disrupt | Neighbor noise disrupt | Delayed rent/mortgage | Food Stamp enrollment | Less high school education | Unemployed | Poverty | % Non-White | % African American | % Hispanic |

**FIGURE CAPTIONS**

**Figure S1:** Kernel density plots comparing pollutant (wintertime PM_2.5_ and SO_2_, and summer O_3_) distributions, by UHF (row 1) and areas reformulated to UHF from other administrative units (rows 2-4).
